# Bacterial Toxin and Effector Regulation of Intestinal Immune Signaling

**DOI:** 10.3389/fcell.2022.837691

**Published:** 2022-02-16

**Authors:** Patrick J. Woida, Karla J. F. Satchell

**Affiliations:** Department of Microbiology-Immunology, Northwestern University Feinberg School of Medicine, Chicago, IL, United States

**Keywords:** toxins, effector, innate, MAP kinasae signaling, NF-kB, nuclear factor-kB, GTPase, therapeutics

## Abstract

The host immune response is highly effective to detect and clear infecting bacterial pathogens. Given the elaborate surveillance systems of the host, it is evident that in order to productively infect a host, the bacteria often coordinate virulence factors to fine-tune the host response during infection. These coordinated events can include either suppressing or activating the signaling pathways that control the immune response and thereby promote bacterial colonization and infection. This review will cover the surveillance and signaling systems for detection of bacteria in the intestine and a sample of the toxins and effectors that have been characterized that cirumvent these signaling pathways. These factors that promote infection and disease progression have also been redirected as tools or therapeutics. Thus, these toxins are enemies deployed to enhance infection, but can also be redeployed as allies to enable research and protect against infection.

## Introduction

Host-pathogen interactions are commonly described as an arms race. Upon detection of a pathogen, the host mounts an immune response. In turn, pathogens fight these responses with tools such as toxins and effectors. For bacterial pathogens to effectively infect and colonize the intestine, the surveillance systems that protect the intestinal tissue need to circumvented. Many intestinal pathogens successfully evade clearance by manipulating the host cell signaling pathways that control initiation of the innate immune program. The evasion strategies include delivery of toxins and effectors into cells and these effectors act to cleave, modify, or circumvent critical proteins in innate immune signaling, resulting in bacterial colonization and successful infection. In turn, the host can detect some of these events to re-initiate the response. This review of published literature will detail the pathways critical for innate immune signaling and summarize the mechanisms of several effectors of intestinal pathogens known to interfere with the host signaling.

## Intestinal Epithelial Cell Strategies to Protect Against Pathogenic Bacteria

The intestinal epithelial cell (IEC) surface itself acts as the first line of defense from invading pathogens. This layer of IECs acts simply as a physical barrier keeping microbes, and other possibly harmful antigens, in the intestinal lumen and by preventing access to the rest of the body (Perez-Lopez et al., 2016). The second line of defense is rapid motility from intestinal villi, in part by normal gut peristalsis, which creates a consistent flow of intestinal contents through the lumen and reduce bacterial growth on the epithelial surface (Santaolalla et al., 2011; Kim et al., 2016). The next barrier is the layer of mucus that covers the surface of the IECs. Mucus is composed of *O*-glycosylated glycoproteins that are primarily secreted by goblet cells and form a gel-like matrix over the cell surfaces (Johansson and Hansson, 2016). Mucus acts to reduce microbial motility and to prevent pathogens from reaching and colonizing the IEC surfaces (Smith et al., 1995; McAuley et al., 2007; Bergstrom et al., 2010; Desai et al., 2016). Mucus also contains a high concentration of antimicrobial peptides, such as α-defenisin and cathelicidin, to clear invading pathogens trapped in the mucus layer (Meyer-Hoffert et al., 2008; Antoni et al., 2013). Since the mucus layers inhibit bacteria from reaching the IEC surface, pathogens must bypass this innate immune barrier, for example, by secreting mucinases (Shon et al., 2021).

Since pathogens can often bypass the mucus layer and colonize the intestinal surface, IECs along with all resident innate immune cells utilize germ-line encoded pattern recognition receptors (PRRs) to detect pathogen/microbial-associated molecular patterns (PAMPs/MAMPs) of the invading pathogens. There are five families of PRRs: Toll-like receptors (TLRs); Nucleotide-binding domain, leucine rich-repeat (LRR)-containing (or NOD-like) receptors (NLRs), RIG-I like receptors (RLRs); and the absent in melanoma (AIM2)-like receptors (ALRs) (Brubaker et al., 2015). These receptors are compartmentalized in the plasma membrane, endocytic vesicles, or the cytosol to detect a broad range of pathogens. PRR detection of a particular PAMP leads to activation of downstream signaling pathways to induce gene expression (Brubaker et al., 2015).

There are 10 members of the human TLR family. Almost all are expressed in colonic IECs, while only TLR1-5, and 9 have been detected in the small intestine. TLRs recognize a diverse array of PAMPS to alert the host to an invading pathogen. TLR1, 2, and 4-6 specifically recognize components of bacterial outer surfaces and are primarily localized at the cell surface, with the exception of TLR4, which can also localize to endosomes (Kawai and Akira, 2006). TLR1 forms heterodimers with either TLR2 or TLR6 to detect triacylated or diacylated lipoproteins respectively (Takeda et al., 2002; Shimizu et al., 2007). TLR4 recognizes lipopolysaccharide (LPS), in combination with adapter proteins CD14, LPS-binding protein (LBP), and myeloid differentiation factor-2 (MD-2) (Poltorak et al., 1998; Kim et al., 2007; Park et al., 2009). TLR5 recognizes bacterial flagellin (Hayashi et al., 2001).

TLR3 and TLR7-9 recognize nucleic acids from bacteria or viruses and are primarily localized to endosomes (Kawai and Akira, 2006). TLR3 binds to double-stranded RNA (Liu et al., 2008) while, TLR7 and 8 bind to single-stranded RNA (Diebold et al., 2004; Heil et al., 2004). TLR9 recognizes bacterial DNA (Hemmi et al., 2000). There are currently no known targets of TLR10.

**FIGURE 1:**
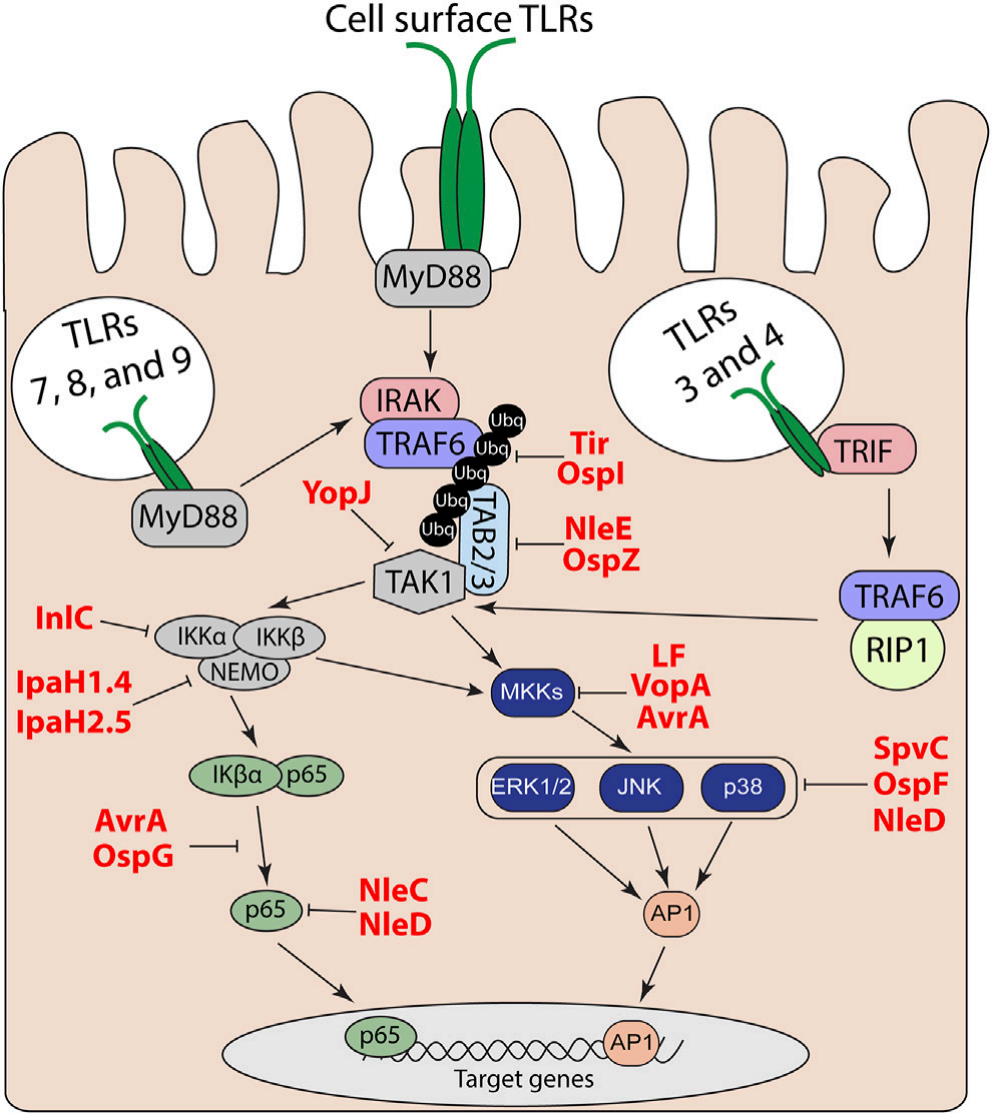
TLR and MAP kinase/NF-kB signal transduction pathways. Effector regulation of TLR signaling through inhibition of MAP kinase and NF-κB pathways to suppress downstream proinflammatory target genes.

TLRs are comprised of an N-terminal LRR ligand recognition domain, a transmembrane domain, and a cytosolic Toll IL-1 receptor (TIR) domain (Botos et al., 2011). Following recognition of a TLRs specific ligand, the TIR domain interacts with other cytosolic TIR domain containing adapter proteins to trigger a signaling cascade that leads to the activation of mitogen-activated protein (MAP) kinases and nuclear factor-kappa B (NF-κΒ) pathways; resulting in the upregulation of proinflammatory genes (Kawai and Akira, 2006). All TLRs, except TLR3, utilize the adapter myeloid differentiation primary response protein 88 (MyD88) (Kawai and Akira, 2006). TLR2 and 4 also utilize the TIR domain-containing adapter protein (TIRAP or MyD88 adapter-like) as an additional adapter for MyD88-dependent signaling (Kawai and Akira, 2006). After TLR activation, the interleukin 1 receptor-associated kinase (IRAK) family of proteins and tumor necrosis factor (TNF) receptor-associated factor 6 (TRAF6) (Cao et al., 1996) are recruited to MyD88 (Cao et al., 1996; Ferrao et al., 2014). The transforming growth factor-β activated kinase (TAK1)-binding proteins 2 and 3 (TAB2 and TAB3) bind to ubiquitin chains on TRAF6 and recruit transforming growth factor-β activated kinase (TAK1) (Kanayama et al., 2004). Endosomal TLR3 and TLR4 utilize TRIF instead of MyD88 to activate TAK1 through TRAF6 and receptor-interacting serine/threonine-protein kinase 1 (RIP1) (Brubaker et al., 2015). The subsequent recruitment of TAK1 leads to phosphorylation and activation of the MAP kinases, specifically extracellular-signal-related kinase (ERK), c-Jun N-terminal kinase (JNK), and p38 MAP kinase. TAK1 also regulate degradation of the NF-κB inhibitor IκBα to allow p50-p65/RelA to translocate to the nucleus and to activate expression of proinflammatory cytokine and chemokine genes (Hacker and Karin, 2006; Liu et al., 2017). TLR activation of NF-κB and MAP kinase signaling via MyD88 and TRIF is outlined in Figure 1.

Given the complexity of the host response to bacterial PAMPS, it is not surprising that bacterial pathogens have likewise elaborate strategies to escape host detection. These strategies include evasion of detection by direct modification of their PAMPs to bypass host detection by TLRs (Paciello et al., 2013). Another approach is to directly manipulate the host to subvert or actively suppress host innate immune signaling. These factors can be extracellularly secreted protein toxins or delivered as effector proteins of direct injection secretion systems. Among many strategies, a number of the effectors function to shut off either MAP kinase or NF-κB pathways to prevent upregulation of proinflammatory cytokines and chemokines (Table 1).

**TABLE 1:** Toxins and effectors that inhibit MAPK and NF-κΒ pathways.

Bacteria	Toxin/Effector	Target	Mechanism	Citation
Inhibition of MAPK signaling				
*Bacillus anthracis*	Lethal Factor (LF)	MKKs	Proteolytic cleavage of N-terminal substrate binding domain of MKK	Park et al. (2002)
*Salmonella Typhimurium*	SpvC	p38, JNK, and ERK 1/2	Phosphothreonine lyase, removes phosphorylated threonine residues	Mazurkiewicz et al. (2008)
*Shigella flexneri*	OspF	p38, JNK, and ERK 1/2	Phosphothreonine lyase, removes phosphorylated threonine residues	Li et al. (2007)
*Vibrio cholerae*	ABH	PtdIns3P	Cleaves PtdIns3P to inhibit autophagy	(Agarwal et al., 2015)
*Vibrio cholerae*	RID	Rho GTPases	Acylates lysine residues in the polybasic region of Rho GTPases	(Zhou et al., 2017)
*Vibrio vulnificus*	RRSP	Ras/Rap1	Cleaves the between Tyrosine-32 and Aspartate-33 to disengage GEF interactions and GTPase activity	(Antic et al., 2015; Biancucci et al., 2018)
*Vibrio parahaemolyticus*	VopA	MKKs	Acetyltransferase, acetylates activation loop of MKKs	(Trosky et al., 2007)
Inhibition of NF-κΒ Signaling				
EPEC	NleB	GAPDH	O-GlcNAc transferase to inhibit GAPDH-TRAF2 interaction	(Gao et al., 2013)
EPEC	NleC	p65/RelA	Zinc protease that degrades p65/RelA transcription factor	(Yen et al., 2010)
EPEC	NleE	TAB2/3	Methylates TAB2/3 NFZ domains and block ubiquitin binding	Newton et al. (2010)
*Listeria monocytogenes*	InlC	IKKα	Binds IKKα and blocks IKK complex from phosphorylating IκΒα	(Gouin et al., 2010)
*Shigella flexneri*	IpaH1.4 and 2.5	HOIL-1L (NF-κΒ E3 Ligase subunit)	E3 ligase activity that promotes HOIL-1L degradation	(de Jong et al., 2016)
*Shigella flexneri*	OspG	Unknown	Binds UbcH7 (E2 ligase) and reduces IκΒα degradation	(Kim et al., 2005)
*Shigella flexneri*	OspZ	TAB2/3	Methylates TAB2/3 NFZ domains and block ubiquitin binding	(Zhang et al., 2016)
Inhibition of both MAPK and NF-κΒ Signaling				
*Salmonella Typhimurium*	AvrA	MKK6/7 and IκΒα	Acetyltransferase for MKK6/7 and deubiquitinase against IκΒα	(Ye et al., 2007; Jones et al., 2008)
EPEC	Tir	SHP-1	Recruit SHP-1 *via* ITIM domain to block TRAF6 ubiquitination	(Yen et al., 2012)
EPEC	NleD	p65/RelA and JNK	Proteolytic cleavage of p65 and JNK	(Baruch et al., 2011)
*Shigella flexneri*	OspI	Ubc13 (E2 ligase)	Deamidase, inhibits Ubc13 activity blocking ubiquitination and activation of TRAF6	(Sanada et al., 2012)
*Vibrio parahaemolyticus*	VopS	Rac1, RhoA, CDC42	AMPylates Threonine-31 to block interaction with downstream Rho effecors	(Woolery et al., 2014)
*Yersinia* sp*.*	YopJ	MEK2, IKK, and TAK1	Acetyltransferase activity	(Mittal et al., 2006)

## Bacterial Toxin and Effector Direct Inhibition of MAP Kinase Signaling

TLR induction of proinflammatory responses is propagated through MAP kinase kinases (MKKs) to activate ERK, p38, and JNK MAP kinase signaling pathways. Many bacterial toxins and effectors have been characterized that directly alter proteins in this pathway (Table 1).

*Bacillus anthracis*, the causative agent of anthrax, secretes anthrax lethal toxin, an AB-subunit toxin that consist of the catalytic lethal factor (LF) and the receptor binding and delivery protein protective antigen (PA) (Bradley et al., 2001). LF is a protease that cleaves the N-terminus of MKKs, separating the N-terminal substrate recognition domain from the C-terminal catalytic domain (Duesbery et al., 1998; Vitale et al., 2000). LF-mediated cleavage inactivates MKKs thereby reducing phosphorylation of ERK, JNK, and p38 (Duesbery et al., 1998; Park et al., 2002). By blocking activation of the MAP kinases, LF induces apoptosis and suppresses cytokine production in response to *B. anthracis* peptidoglycan (Park et al., 2002; Barua et al., 2013).

While LF cleaves MKKs, multiple type III secretion (T3S) effectors inactive MKK activity through acetylation. VopA from *Vibrio parahaemolyticus* inactivates MKKs by acetylating four conserved residues in the catalytic loop of MKKs to block ATP binding (Trosky et al., 2004; Trosky et al., 2007). Both AvrA from (Baruch et al., 2011) *Salmonella enterica serovar Typhimurium* (*S. Typhimurium*) and YopJ from *Yersinia* spp. also act as acetyltransferases to inhibit MKKs, but instead acetylate residues that are necessary for normal phosphorylation of the MKK and thus block their activation (Mittal et al., 2006; Jones et al., 2008). However, while VopA targets MKKs associated with ERK, JNK, and p38 MAP kinase pathways, AvrA has specific activity towards MKK4 and 7, which preferentially activate JNK. Additionally, YopJ targets MEK2 and TAK1, which regulate ERK signaling (Mittal et al., 2006; Trosky et al., 2007; Jones et al., 2008).

In addition to targeting MKKs, some bacteria can actively remove phosphate groups from the MAP kinases to directly inactive these signaling kinases. *Shigella flexneri* T3S effector OspF, and its homolog in *S. typhimurium* SpvC are phosphothreonine lyases that remove phosphate from the threonine residues required for MAP kinase activation (Trosky et al., 2007; Zhu et al., 2007; Mazurkiewicz et al., 2008). The benefit of the phospholyase reaction, instead of a traditional phosphatase reaction, is that OspF/SpvC removes the hydroxyl-group and forms a C=C bond on the threonine residue. Thus, OspF/SpvC permanently inactive the MAP kinase by preventing re-phosphorylation and reactivation of the MAP kinase pathway by the host (Li et al., 2007; Mazurkiewicz et al., 2008). SpvC dephosphorylates ERK1/2 in mouse IEC mucosa to suppress expression of genes for macrophage inflammatory protein 2, TNF, CXCL1, and other proinflammatory cytokine and chemokine genes to reduce neutrophil recruitment during the early stages of infection (Haneda et al., 2012). It is important to note that SpcV only reduces and does not completely abolish the proinflammatory response. This suggests SpcV is not sufficient to abolish inflammation or SpvC is temporally regulated since inflammation is beneficial to *S. Teyphimurium* metabolism during infection (Spiga et al., 2017).

## Suppression of NF-κB Signaling by Bacterial Toxins and Effectors

Pathogen suppression of host innate immune signaling is not restricted to the MAP kinase pathway. During TLR signaling, TRAF6 undergoes autoubiqutination to recruit TAB2/3 and TAK1 to activate IκB kinase (IKK) and downstream NF-κB signaling (Kanayama et al., 2004). Enteropathogenic *Escherichia coli* (EPEC) and *S. flexneri* secrete the T3S effectors NleE and OpsZ to methylate cysteine residues in the TAB2/3 Npl4 zinc finger (NFZ) domains and block NFZ-mediated TAB2/3 binding to ubiquitin (Zhang et al., 2011; Zhang et al., 2016). NleE/OspZ methylation of TAB2/3 blocks p65/RelA translocation to the nucleus and inhibits expression of *CXCL8* that encodes the neutrophil chemokine IL-8 (Newton et al., 2010; Zhang et al., 2016). TRAF2 can form a complex with glyceraldehyde 3-phosphate dehydrogenae (GAPDH) to recruit TAB2/3 and activate TAK1 independently of TRAF6 (Gao et al., 2013). EPEC utilizes NleB as a N-acetylglucosamine (GlcNAc) transferase to add O-GlcNAc to GAPDH and block the TRAF2-GAPDH interaction and inhibit TAK1 induction of NF-κB (Gao et al., 2013). In addition to acetylating MKKs, YopJ from *Yersinia* spp. also acetylates both IKKα and IKKβ, which block their ability to phosphorylate IκΒα (Mittal et al., 2006). This forces IκΒα to retain p65/RelA in the cytosol and prevents activation of NF-κB regulated-proinflammatory genes (Mittal et al., 2006). *Listeria monocytogenes* also directly targets the IKK complex through intracellular secretion of internalin C (InlC), which directly binds IKKα and inhibits formation of the active IKK complex to suppress the early NF-κB response to *L. monocytogenes* infection (Gouin et al., 2010).

Since TLR signaling is tightly regulated by ubiquitination and activation of the proteasome (Hu and Sun, 2016), many bacterial toxins and effectors target instead the host ubiquitination machinery to modulate NF-κB signaling. For example, in addition to its acetyltransferase function, AvrA also has deubiquitinase activity and removes ubiquitin from IκΒα, thereby preventing its degradation resulting of retention of p65/RelA in the cytosol (Ye et al., 2007). Therefore, AvrA can suppress both MAP kinase and NF-κB signaling through entirely different mechanisms.

The *S. flexneri* effector OspG also inhibits IκΒα degradation by binding to the E2 conjugating enzyme Ubc5Hb, which is required for ubiquitination of IκΒα. While OspG autophosphorylation activity and binding to Ubc5Hb were required to block IκΒα ubiquitination and degradation, it was not found to modify UbcH5b (Kim et al., 2005). Therefore, whether OspG physically blocks E1 enzymes from ubiquitinating UbcH5b or OspG modifies other factors required for these processes still needs to be determined. Deletion of *ospG* in *S. flexneri* led to significant increase in inflammation in the rabbit ileal loops revealing that OspG dampens the host response to infection (Kim et al., 2005).

Linear ubiquitination of the NF-κB essential modulator (NEMO) by the linear ubiquitin chain assembly complex (LUBAC) is critical for NEMO activation and phosphorylation of IκΒα (Tokunaga et al., 2009). *S. flexneri* effectors IpaH1.4 and IpaH2.5 are E3 ligases that ubiquitinate the heme-oxidized IRP2 ubiquitin ligase 1 (HOIL-1) subunit of LUBAC to promote its proteasomal degradation (de Jong et al., 2016). Degradation of HOIL-1 then prevents linear ubiquitination of NEMO by LUBAC and thus inhibits IκΒα phosphorylation, degradation, and NF-κB activation. IpaH1.4 targeted degradation of HOIL-1 also blocks ubiquitination of the bacterial surface and prevents autophagic clearance of cytosolic bacteria (Noad et al., 2017). While both IpaH1.4 and IpaH2.5 share identical biochemical function, only deletion of *ipaH1.4* abolishes HOIL-1 degradation and promotes translocation of p65/RelA to the nucleus (de Jong et al., 2016). p65/RelA also translocated to the nucleus in cells infected with *S. flexneri* that had a deletion in the gene of another T3S effector *ospI* (de Jong et al., 2016) revealing that both IpaH1.4 and OspI target NF-κB signaling through non-redundant mechanisms. OspI had been previously identified as an enzyme that deamidates glutamine-100 of the E2 conjugating enzyme UBC13 (Sanada et al., 2012). Deamidation of Q100 impairs UBC13 E2 activity required for TRAF6 autoubiquitination and thus prevents TRAF6 activation. Since TRAF6 activation is required for both NF-κB and MAP kinase activation, OspI deamidase activity was also shown to inhibit JNK signaling (Sanada et al., 2012). Microarray analysis of wild type and *Δospi* infected HeLa cells revealed that OspI suppresses expression of *TNF*, *CXCL8*, *IL6* and other proinflammatory genes induced during *S. flexneri* infection (Sanada et al., 2012).

## Control of Inflammatory MAP Kinase Pathways by *Vibrio*s Through Inhibition of Small GTPases

New studies of effectors delivered by *Vibrios* have unveiled additional strategies for control of MAP kinase and NF-κB signaling that is, directed through inhibition of host small GTPases. The cellular small GTPases cycle between an inactive guanine nucleotide diphosphate (GDP)-bound state and an active guanine nucleotide triphosphate (GTP)-bound state. The activation states are tightly regulated by guanine nucleotide exchange factors (GEF) that exchange bound GDP with GTP and by GTPase activating proteins (GAPs) that induce the GTP hydrolysis activity to revert them to the inactive GDP-bound state (Hodge and Ridley, 2016). Many of the GTPase also utilize a C-terminus lysine/arginine rich poly basic region (PBR) along with additional post translational modifications to localize to distinct regions of the plasma membrane to promote GTPases activity (Roberts et al., 2008; Navarro-Lerida et al., 2012; Hodge and Ridley, 2016).

The best characterized small GTPases linked to pathogenesis are the Ras and Rho family GTPases. The Ras family GTPases directly control MAP kinases downstream of epidermal growth factor receptor (EGFR) with activated Ras recruiting the kinase RAF that then phosphorylates MEK leading to ERK phosphorylation. By contrast, the Rho family GTPases are best characterized as promoting actin fiber assembly and stabilization. During infection, inhibition of small GTPases by bacterial toxins and effectors has centered on their role in control of actin with inhibition of the Rho family GTPases resulting in loosen tight junctions between IEC and inhibition of phagocytosis by macrophages (Aktories and Barbieri, 2005). However, the Ras and Rho family GTPases are also recognized as critical regulators of inflammation suggesting they may have a significant role in pathogenesis unrelated to the cytoskeleton.

*Vibrio cholerae* recently was shown to have potential to induce a potent proinflammatory immune response though its destruction of polymerized actin via the actin crosslinking effector domain (ACD) of its secreted multifunctional-autoprocessing repeats in toxin (MARTX) toxin. Indeed, destruction of actin was found itself to be a damage-associated molecular pattern (DAMP) that induces the MAP kinase pathways resulting in phosphorylation of ERK, p38, and JNK (Woida and Satchell, 2020). Upregulation of MAP kinase signaling was shown to be driven through the Rho family GTPase Rac1 (Woida and Satchell, 2020). However, the mechanistic link between the depolymerization of actin and activation of Rac1 and downstream MAP kinase pathways has not been yet elucidated.

Yet, despite this potential to activate Rac1 and the MAP kinase pathways, the MARTX toxin of *V. cholerae* does not induce a proinflammatory immune response because another effector domain delivered by the MARTX toxin, the Rho GTPase inactivation domain (RID) silences signal flow through the MAP kinase pathway (Woida and Satchell, 2020). RID functions by acylating lysine residues, primarily Lys-186 and Lys-188, in the PBR of Rac1 to inhibit its function as a GTPases (Zhou et al., 2017). RID also targets RhoA and CDC42, but has preference for Rac1 (Zhou et al., 2017). Rac1 is known to activate MAP kinases particularly through p21-activated kinase 1 (PAK1). Thus, the acylation of Rac1 by RID silences the immune response to *V. cholerae* by inhibition of MAP kinase signaling (Woida and Satchell, 2020).

The *V. cholerae* MARTX toxin also co-delivers the α/β hydrolase (ABH) effector domain to block ACD induced inflammation. ABH is a phosphatidylinositol-3-phosphate (PtdIns3P) specific phospholipase that blocks ACD mediated activation of Rac1 and downstream MAP kinase signaling (Agarwal et al., 2015; Woida and Satchell, 2020). ABH cleavage of PtdIns3P also inhibits autophagy and upregulates CDC42 (Agarwal et al., 2015; Dolores et al., 2015). Mutations in autophagy genes such as *ATG16L1* causes defects in granule exocytosis of Paneth cells impairing secretion of antimicrobial products (Cadwell et al., 2009). However, defects in Paneth cell autophagy often leads to impairments in the unfolded protein response (UPR) causing endoplasmic reticulum stress (ERS), upregulation of proinflammatory genes through Rho GTPases, and contributes to the development of irritable bowel disease (Wang et al., 2018). During *V. cholerae* infection, ABH inhibition of autophagy in Paneth cells could block secretion of antimicrobial products and promote bacterial colonization. However, while this would trigger ERS/UPR dependent inflammation, co-delivery of RID would further inhibit this response. Therefore, co-delivery of ACD, RID, and ABH on the same toxin allows *V. cholerae* to damage host tissue without eliciting an inflammatory response by tightly regulating small GTPases.

Curiously, the MARTX toxin of some *Vibrio vulnificus* isolates also have an and these strains have been shown to be potently proinflammatory to set off a cytokine storm (Murciano et al., 2017). Several newly identified strains with an ACD have recently been identified that also carry the Ras/Rap-specific endopeptidase (RRSP) effector domain (Lee et al., 2019). This effector domain cleaves RAS between Tyr-32 and Asp-33 to disengage the GTPase from the GEF that activates GTP exchange and to halt GTPase activity (Antic et al., 2015; Biancucci et al., 2018). The inactivation also prevents RAS interaction with RAF kinases to inhibit downstream phosphorylation of ERK (Biancucci et al., 2018). Indeed, this effector has been demonstrated to be a potent inhibitor of ERK phosphorylation in colonic epithelial cells (Stubbs et al., 2021). Thus, we speculate that RRSP, like RID, may also manipulate MAP kinase signaling to control the proinflammatory immune response to promote disease progression (Gavin et al., 2017).

*Vibrio parahaemolyticus* has also been shown to induce inflammation via the T3S effector VopQ that is injected into epithelial cells. VopQ interacts with the V_0_ domain of the H^+^-ATPase to form pores in the ATPase associated membranes to block autophagic turnover and protects the bacteria from macrophage phagocytosis (Burdette et al., 2009; Sreelatha et al., 2013). In addition, this effector forms pores in the endoplasmic reticulum (ER) membrane resulting in activation of the unfolded protein response through the inositol-requiring enzyme 1 (IRE1) kinase pathway (De Nisco et al., 2021). The activation of IRE1 results in downstream activation of MAP kinases, including ERK. Thus, ER damage induced by VopQ is easily detected by the cells, resulting in MAP kinase activation. However, the parallel delivery of another T3S effector VopS ameliorates the MAP kinase activation. The *V. parahaemolyticus* T3S effector VopS blocks RhoA, Rac1, and CDC42 from interacting with their downstream effectors by transferring an adenosine monophosphate to Thr-31, a process known as AMPylation (Yarbrough et al., 2009). Interestingly, unlike RID, VopS also inactivates downstream NF-κB signaling (Woolery et al., 2014; Woida and Satchell, 2020). This difference may be attributed to substrate specificity since VopS targets RhoA, Rac1, and CDC42 equally while RID has preference for Rac1 (Yarbrough et al., 2009; Woida and Satchell, 2020) or be may be due to the mechanistic difference of RID modifying the PBR as opposed to VopS modification of the Switch I region.

## Regulation of the Inflammasome and Fighting Back Against Effector Triggered Immunity

**FIGURE 2:**
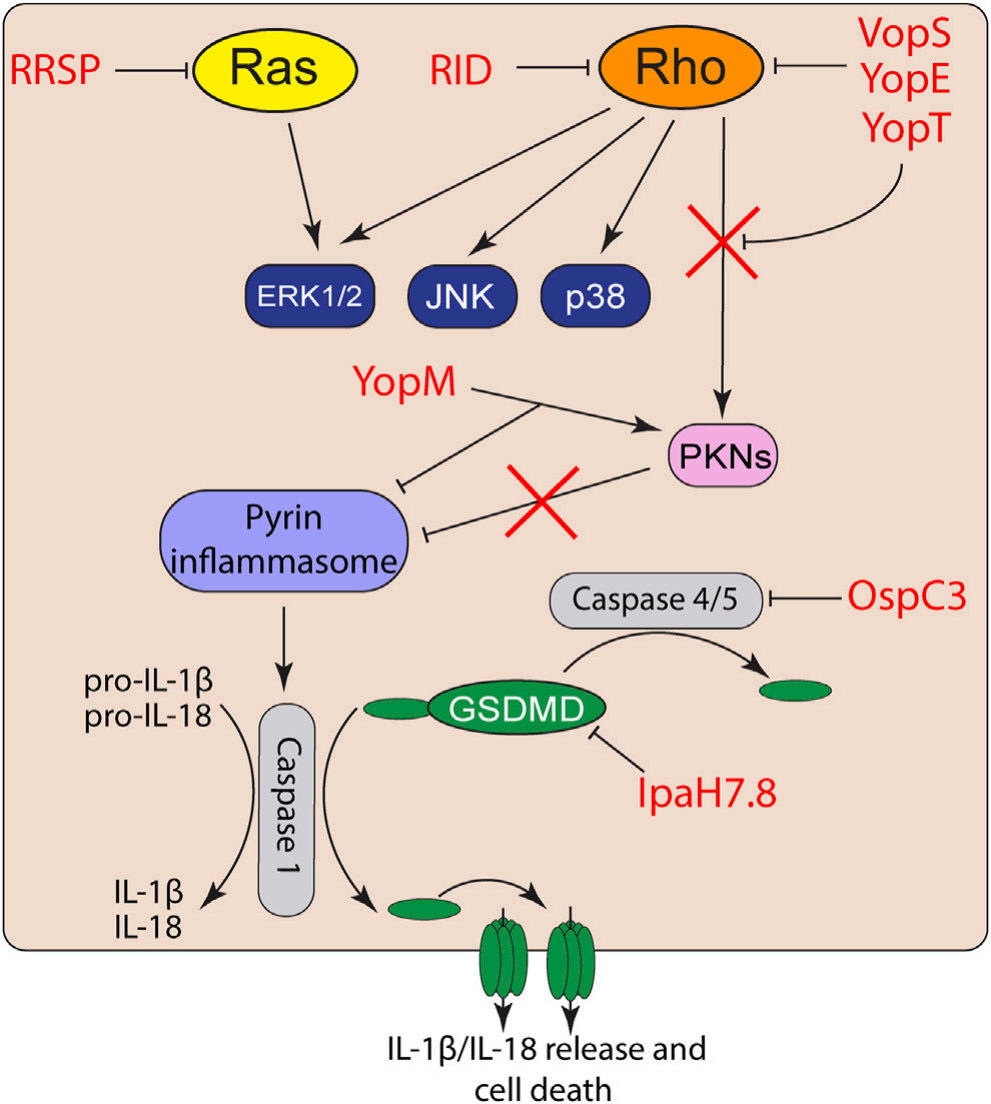
Effector activation and suppression of effector triggered immunity. Toxin and effector targeting of Rho GTPases trigger activation of the pyrin inflammasome. However, bacterial pathogens also co-deliver effectors to suppress inflammasome activation or inactivate proinflammatory caspases and gasdermins.

While bacteria have an arsenal of tools to suppress detection of PAMPs, host cells can detect this activity or the damage the toxins induce. NLRs, AIM2, and pyrin are cytosolic PRRs that function not only to detect pathogens, but can also directly detect toxin activity to trigger inflammasome activation (Schnappauf et al., 2019; Zheng et al., 2020) (Figure 2). Inflammasomes cleave and activates pro-caspase-1, which subsequently cleaves pro-IL-1β and pro-IL-18 leading to their activation as highly proinflammatory cytokines (Martinon et al., 2002; Zheng et al., 2020). Caspase-1 also cleaves the N-terminal of gasdermin-D (GSDMD) (Sborgi et al., 2016), which oligomerizes to form large non-specific pores in the cell membrane that induce cell death and the release of intracellular IL-1β/IL-18 (Shi et al., 2015; Sborgi et al., 2016).

While VopS suppresses activation of MAP kinase and NF-κB signaling in epithelial cells (Yarbrough et al., 2009; De Nisco et al., 2021), AMPylation of Rho GTPases by VopS activates the pyrin inflammasome (Xu et al., 2014). The pyrin inflammasome is also activated in response to toxin/effector-mediated Rho GTPase glycosylation, deamidation, and proteolytic cleavage (Xu et al., 2014). Activated RhoA normally turns on protein kinase C-related kinases (PKNs) that phosphorylate pyrin. The phosphorylation of pyrin recruits 14-3-3 to inhibit pyrin inflammasome activation (Chung et al., 2016). However, inactivation of RhoA by toxins and effectors inactivate PKN activity and thus prevents pyrin phosphorylation and subsequently induce the pyrin inflammasome (Xu et al., 2014; Schnappauf et al., 2019). Curiously, acylation of Rho GTPases by the MARTX effector RID does not trigger inflammasome activation (Xu et al., 2014). How RID acylation avoids effector triggered immunity is not known but is likely related to its modification of the PBR rather than the GTPases Switch I region.

Similar to many *Vibrio* species, *Yersinia* also targets Rho GTPases using two different T3S effectors. YopE is a GAP mimic that induces Rho GTP-hydrolysis and forces Rho GTPases to their inactivate GDP-bound form (Von Pawel-Rammingen et al., 2000). YopT is a cysteine protease that cleaves the N-terminus of RhoA to detach the GTPase from the plasma membrane and block interaction with its downstream effectors (Shao et al., 2003). While both YopT and YopE induce actin depolymerization and block phagocytosis (Shao et al., 2003), both independently trigger inflammasome activation (Chung et al., 2016). However, *Yersinia* also delivers YopM, which binds to PKNs and recruits them to pyrin to maintain pyrin phosphorylation (Chung et al., 2016). Co-delivery of YopM with YopE and YopT allows *Yersinia* to inactivate Rho GTPases without triggering the inflammasome.

In addition to detecting toxin modification of Rho GTPases, cells can also detect bacterial-mediated MAP kinase suppression. For example, while the *B. anthracis* LF protease suppresses MAPK signaling, it also cleaves a portion of the N-terminus of the cytosolic PRR NLRP1B, which activates the inflammasome (Chavarria-Smith and Vance, 2013). NLRP1B naturally undergoes autoprocessing to release its C-terminal CARD domain, which remains non-covalently attached to the remaining protein (Frew et al., 2012). By contrast, cleavage of NLRP1B by LF exposes the N-terminus to proteasomal degradation and releases the non-covalently attached C-terminal CARD domain, resulting in assembly of an active NLRP1 inflammasome (Frew et al., 2012; Chavarria-Smith and Vance, 2013). NLRP1 inflammasome activation promotes neutrophil recruitment and protects against *B. anthracis* in mice (Moayeri et al., 2010).

NLRP1B activation also detects the E3 ubiquitin ligase activity of the *S. flexneri* T3S effector IpaH7.8. Instead of cleaving NLRP1B, IpaH7.8 directly ubiquitinates the N-terminus, thus targeting the protein for proteasomal degradation and releasing the bioactive C-terminal CARD domain (Sandstrom et al., 2019). However, IpaH7.8 targets only mouse, and not human, NLRP1B (Luchetti et al., 2021). Instead, IpaH7.8 ubiquitinates human GSDMD for degradation and blocks NLRC4 and caspase-mediated pyroptosis (Luchetti et al., 2021). Interestingly, IpaH7.8 has also recently been shown to ubiquitinate human gasdermin B (GSDMB) (Hansen et al., 2021). Unlike GSDMD, GSDMB targets lipids enriched in bacterial membranes to directly form pores and lyse intracellular bacteria during infection (Hansen et al., 2021). GSDMB is also not activated by caspases, but instead is activated by granzyme B released into epithelial cells by natural killer cells recruited to the site of infection (Hansen et al., 2021).

Activation of GSDMD can also be activated independently of inflammasome signaling. Detection of cytosolic LPS by caspases-4/5 (and caspase-11 in mice) leads to caspase mediated cleavage and activation of GSDMD (Broz et al., 2020). *S. flexneri* T3S effector OspC3 further suppresses GSDMD activation through ADP-riboxanation of Arg-314 and Arg-310 of caspases 4 and 11, respectively, block caspase-4 and 11 autoprocessing and activation of GSDMD (Li et al., 2021). The dual targeting of the gasdermin family members by IpaH7.8 and OspC3 allows *S. flexneri* to both block inflammasome driven inflammation and direct bacterial killing during intestinal infection.

## Hijacking Immune Targeting Toxins for Cancer Therapeutics

As the role of many bacterial toxins is to suppress immunity and inhibit inflammation, with the goal of promoting infection, bacterial toxins are most commonly detrimental to host health and survival. However, when removed from the context of the pathogen, some bacterial toxins and effectors have proven advantageous when deployed as therapeutics.

In addition to regulating innate immune signaling, MAP kinase signaling regulates cell growth and differentiation (Rodriguez-Viciana et al., 2004). Hyperactivation of Ras GTPases and their downstream signaling networks, such as the MAP kinase pathways, leads to uncontrolled cell growth and tumorigenesis (Schubbert et al., 2007). More than one-third of all human cancers contain mutations in *RAS* or genes in their downstream signaling pathways that promote tumor survival (Hobbs et al., 2016). This high prevalence demonstrates a critical need to develop new therapeutics to treat Ras driven tumors.

The discovery of the RRSP MARTX toxin effector (as described above) presented the potential of repurposing RRSP as a biologic to target Ras driven tumors. Recombinant RRSP was fused to the translocation/receptor binding domain of diphtheria toxin (DT_B_) and this engineered chimeric toxin RRSP-DT_B_ was shown to cleave RAS, decrease MAP kinase phosphorylation, and inhibit proliferation of a wide spectrum of cancer cells (Vidimar et al., 2020). This reduction in growth is a result of RRSP inducing cells to enter p27-mediated cell cycle arrest (Stubbs et al., 2021) and its inhibition of cyclin dependent kinase 1 (Wang et al., 2020). As DT_B_ binds to its receptor human heparin-binding epidermal growth factor-like growth factor (HB-EGF) at 1000-fold greater affinity compared to mouse HB-EGF (Palmiter, 2001), intraperitoneal injected RRSP-DT_B_ preferentially targets human tumors in xenograft mice. When mice with breast or colon cancer xenograft tumors are treated with RRSP-DT_B_, the tumors were significantly reduced in size (Vidimar et al., 2020). Immunostaining of recovered tumors showed cleavage of Ras and inactivation of downstream ERK MAP kinase signaling (Vidimar et al., 2020). Altogether, these studies demonstrate how RRSP can be employed as a Ras specific cancer therapeutic to induce cell cycle arrest and reduce growth of human tumors.

While DT_B_ provides an excellent proof-of-concept system to target human tumor xenografts in mice, alternate approaches need to be developed for tumor-specific targeting in humans. For example, the recombinant immunotoxin moxetumomab pasudotox has been successfully developed to retarget *Pseudomonas* exotoxin A to bind CD22 on hairy cell leukemia cells to drive cellular apoptosis (Kreitman and Pastan, 2020). In addition, several other toxin-based targeting and delivery strategies have been repurposed to selectively target specific tumor types. For example, overexpression of the human EGFR-2 (HER2) occurs in many breast, gastric, and ovarian cancers (Oh and Bang, 2020). *B. anthracis* PA was specifically modified to inhibit binding to its natural receptors ANTRX1/ANTRX2 and to instead bind to HER2 by attaching PA to an affibody specific for the HER2. This allowed for selective delivery of LF-fused effectors to HER2 positive cells (McCluskey et al., 2013). Similar studies redirected PA to bind exclusively to cells overexpression EGFR by fusing PA to EGF (Mechaly et al., 2012).

A more recent study modified PA by attachment to a single-chain variable fragment antibody to preferentially target pancreatic cancers that overexpress EGFR or the carcinoembryonic antigen (CEA). This system allowed for specific delivery of chimeric proteins with the active domains of RRSP or diphtheria toxin into pancreatic cancer cells (Loftis et al., 2020). Thus, numerous strategies are underway for tumor specific targeting and demonstrate that modified toxin delivery systems could preferential targeting of tumors with effectors that specifically target oncogenic pathways and reduce tumor progression. These strategies could be coupled with any of the effectors that suppress immunity to also potentially treat inflammation diseases.

## Conclusion

Bacterial intestinal pathogens have an arsenal of tools to suppress innate immune pathways to block expression of proinflammatory genes and enhance infection. However, host cells can detect the toxin activity to trigger activation of the inflammasome and gasdermin leading to highly inflammatory mechanisms of cell death. Bacteria have additional tools to then bypass host detection of effector activity or directly modify gasdermin or key regulators of inflammasome activation to suppress the host response to toxin or effector activity. These coordinated actions allow the bacteria to create the optimal environment to promote infection. Since many of these manipulated pathways overlap with those dysregulated in human cancers, bacterial toxins and effectors can be hijacked and employed as potential therapeutics and target the specific pathways upregulated in tumors.

However, there is still significant hurdles in translating the pathogenic potential of bacterial effectors into clinical therapies. Work is progressing to address these hurdles including engineering strategies for chimeric toxins to selectively target cancer cells and to enhance safety and efficacy of these molecules. However, the arsenal of potential effectors that could be used to target diseases associated with MAPK or NF-kB signaling or inflammasome activation (Tables 1, 2) suggest there are many potential strategies for these foes to be repurposed as allies.

**TABLE 2:** Toxin and effectors of intestinal pathogens that regulate Inflammasome and Gasdermin.

Bacteria	Toxin/Effector	Target	Mechanism	Citation
Inflammasome/Gasdermin activators				
*Bacillus anthracis*	Lethal Factor (LF)	NLRP1B	Cleaves and activates proteasomal degradation of the NLRP1B N-terminus to release the C-terminal CARD domain and activate the inflammasome	(Chavarria-Smith and Vance, 2013)
*Shigella flexneri*	IpaH7.8	NLRP1B	E3 Ligase that polyubiquitinates the NLRP1B N-terminus to trigger proteasomal degradation of the NLRP1B N-terminus to release the C-terminal CARD domain and activate the inflammasome	(Sandstrom et al., 2019)
*Yersinia* species	YopE	Rac1, RhoA, CDC42	GAP mimic and induces GTP hydrolysis to forces Rho GTPases into their inactive GDP bound form and triggers pyrin inflammasome	(Von Pawel-Rammingen et al., 2000; Chung et al., 2016)
*Yersinia* species	TopT	Rac1, RhoA, CDC42	Cysteine protease that cleaves the C-terminal PBR and membrane targeting of Rho GTPases and triggers pyrin inflammasome	(Shao et al., 2003; Xu et al., 2014)
*Vibrio parahaemolyticus*	VopS	Rac1, RhoA, CDC42	AMPylates Threonine-31 to block interaction with downstream Rho effectors and triggers pyrin inflammasome	Xu et al. (2014)
Inflammasome/Gasdermin supressors				
*Shigella flexneri*	IpaH7.8	Gasdermin D	E3 Ligase that polyubiquitinates and triggers proteomsomal degradation of GSDMD	Luchetti et al. (2021)
*Shigella flexneri*	IpaH7.8	Gasdermin B	E3 Ligase that polyubiquitinates and triggers proteomsomal degradation of GSDMB	Hansen et al. (2021)
*Shigella flexneri*	OspC3	Caspase-4, -5, -11	ADP-Riboxinates Arg-13 and Arg-310 of caspase-4 and mouse caspase 11 respectively to block caspase autoprocessing and recognition of GSDMD	Li et al. (2021)
*Yersinia* species	YopM	PRK1 and PRK2	Binds and recruits PRK1 and PRK2 to pyrin to maintain pyrin phosphorylation and inactivation	Chung et al. (2016)
